# SRF depletion in early life contributes to social interaction deficits in the adulthood

**DOI:** 10.1007/s00018-022-04291-5

**Published:** 2022-05-04

**Authors:** Matylda Roszkowska, Anna Krysiak, Lena Majchrowicz, Karolina Nader, Anna Beroun, Piotr Michaluk, Martyna Pekala, Jacek Jaworski, Ludwika Kondrakiewicz, Alicja Puścian, Ewelina Knapska, Leszek Kaczmarek, Katarzyna Kalita

**Affiliations:** 1 Laboratory of Neurobiology, Nencki-EMBL Partnership for Neural Plasticity and Brain Disorders – Braincity, Nencki Institute of Experimental Biology, Polish Academy of Sciences, 3 Pasteur Street, 02-093 Warsaw, Poland; 2 Laboratory of Neuronal Plasticity, Nencki-EMBL Partnership for Neural Plasticity and Brain Disorders – Braincity, Nencki Institute of Experimental Biology, Polish Academy of Sciences, 3 Pasteur Street, 02-093 Warsaw, Poland; 3 Laboratory of Molecular and Cellular Neurobiology, International Institute of Molecular and Cell Biology, 4 Ks. Trojdena Street, 02-109 Warsaw, Poland; 4 Laboratory of Emotions Neurobiology, Nencki-EMBL Partnership for Neural Plasticity and Brain Disorders – Braincity, Nencki Institute of Experimental Biology, Polish Academy of Sciences, 3 Pasteur Street, 02-093 Warsaw, Poland

**Keywords:** SRF, Neurodevelopmental disorders, Synapse maturation

## Abstract

Alterations in social behavior are core symptoms of major developmental neuropsychiatric diseases such as autism spectrum disorders or schizophrenia. Hence, understanding their molecular and cellular underpinnings constitutes the major research task. Dysregulation of the global gene expression program in the developing brain leads to modifications in a number of neuronal connections, synaptic strength and shape, causing unbalanced neuronal plasticity, which may be important substrate in the pathogenesis of neurodevelopmental disorders, contributing to their clinical outcome. Serum response factor (SRF) is a major transcription factor in the brain. The behavioral influence of SRF deletion during neuronal differentiation and maturation has never been studied because previous attempts to knock-out the gene caused premature death. Herein, we generated mice that lacked SRF from early postnatal development to precisely investigate the role of SRF starting in the specific time window before maturation of excitatory synapses that are located on dendritic spine occurs. We show that the time-controlled loss of SRF in neurons alters specific aspects of social behaviors in SRF knock-out mice, and causes deficits in developmental spine maturation at both the structural and functional levels, including downregulated expression of the AMPARs subunits GluA1 and GluA2, and increases the percentage of filopodial/immature dendritic spines. In aggregate, our study uncovers the consequences of postnatal SRF elimination for spine maturation and social interactions revealing novel mechanisms underlying developmental neuropsychiatric diseases.

**Supplementary Information:**

The online version contains supplementary material available at 10.1007/s00018-022-04291-5.

## Introduction

Neuronal development is controlled at the transcriptional level. The mutations of genes that encode proteins controlling activity-dependent transcription and chromatin remodeling are frequently associated with neurodevelopmental disorders [1–5]. Disturbances in the genome-wide gene expression throughout the brain development may affect synapse number, structure and strength, leading to disrupted neuroplasticity [6–9], being a common hallmark of different neurodevelopmental diseases, and contributing to their clinical outcome.

Serum response factor (SRF) is a major transcription factor in the brain. The conditional mutant mice with prenatal deletion of SRF have demonstrated that SRF controls neuronal cell migration, neurite outgrowth, hippocampal and cortical lamination, organization of the dentate gyrus (DG), and the formation of mossy fiber circuitry during brain development [10–15]. However, the behavioral consequences of the observed deficits are unknown due to the early animal death [10, 11, 16]. Despite advances in understanding the role of SRF in the central nervous system [17, 18], its contributions to postnatal dendritic spine development, and alterations of complex forms of animals’ behavior in the adulthood remain unresolved.

Social interactions are fundamental and adaptive components of the biology of numerous species, including rodents and humans. Sociability includes some of the most complex behaviors regulated by different patterns of neuronal activation. It involves the detection, consolidation, and interpretation of social cues and processing of context-specific responses that are species-typical [19]. Although sociability impairments are among distinctive and highly disabling features of neurodevelopmental disorders such as fragile X syndrome or autism spectrum disorder (ASD), the molecular mechanism regulating social deficits remains largely unexplained. Addressing biological bases underlying deficits in social behavior requires unbiased and accurate measurements that enable linking molecular/genetic findings with their behavioral manifestations [20]. The conventional tests of social phenotyping has limited cross-laboratory standardization and reproducibility [21]. The new automated Eco-HAB^®^ system enables precise and reliable behavioral characterization of individual animals living in the social groups while controlling confounding factors, and allows better differentiation of disease-specific genotypes [21]. Such accurate social phenotyping is critical to unravel neurobiological underpinnings of brain pathologies.

Here, we investigated the role of SRF in social behavior and glutamatergic synapse maturation by deleting SRF at the early postnatal neuronal development in hippocampal pyramidal neurons in vivo and in vitro. We took a holistic approach to address its impact on behavioral phenotypes of animals living in the groups, and to find a possible molecular cause. We generated mice that lacked SRF in the specific time window before dendritic spine maturation occurs. Our results demonstrate that the time-controlled loss of SRF in hippocampal neurons alters distinct aspects of social behaviors in SRF knock-out (KO) mice, and causes deficits in developmental dendritic spine maturation at both the structural and functional levels. SRF deficiency during the early stages of development decreased the number of functional synapses, downregulated expression of the AMPARs subunits GluA1 and GluA2 and increased the percentage of filopodial/immature dendritic spines. Altogether, our results give a comprehensive view that developmental disturbances of SRF-mediated gene expression impair dendritic spine maturation, which may contribute to long-term consequences for animal’s social behavior in the adulthood. The presented findings implicate developmental SRF activity in the pathogenesis of neurodevelopmental disorders.

## Materials and methods

### Animals

CaMKCreER^T2^, SRF KO (*Srf*^*f/fCaMKCreERT2*^), control CreER^T2^-negative littermates (*Srf*^*f/f*^) or Cre reporter line Ai14 (*loxP*-flanked STOP tdTomato^*CaMKCreERT*2^) were used [22–25]. To achieve the expression of Cre-recombinase in forebrain excitatory neurons that imitate endogenous expression of CaMKIIα, a bacterial artificial chromosome system (BAC) was used. CreER^T2^ fusion construct (Cre recombinase and the mutated ligand-binding domain of the human estrogen receptor) was cloned in the reading frame at the ATG of the CaMKIIα gene present a BAC. This manipulation ensures that Cre-recombinase expression is controlled by the regulatory element of the endogenous CaMKIIα gene. On P5-6, *Srf*^*f/f*^, *Srf*^*f/fCaMKCreERT2*^ or *loxP*-flanked STOP tdTomato^*CaMKCreERT*2^ pups were intraperitoneally injected every other day with three doses of 0.25 mg 4-hydroxytamoxifen (4-OHT; Sigma, #7904) or one dose of 0.75 mg of this compound in sunflower oil. Pups or young adult animals (2–3 months, males and females) were used. The mice were housed in individual cages under a 12 h/12 h light/dark cycle with food and water available ad libitum. The studies were performed in accordance with the European Communities Council Directive of November 24, 1986 (86/609/EEC), Animal Protection Act of Poland and approved by the 1st Local Ethics Committee in Warsaw (permission no 622/2018, 951/2019, 984/2020 and 1193/2021). All efforts were made to minimize the number of animals used and their suffering.

### Eco-HAB^®^ testing of social behaviors

To test social behavior in SRF KO mice, we used Eco-HAB^®^ (patent US10638722) [21], fully automated, RFID-based system that allows the efficient evaluation of animals’ social phenotypes with no contact between tested animals and experimenters (under semi-naturalistic conditions). The group-housed animals (SRF KO and littermate controls, 2–3 months old, genotypes testes separately) were housed under a 12 h/12 h light/dark cycle with unlimited access to food and water. Mice were subjected to the 96-h testing protocol, which consisted of an adaptation phase (72 h) and the subsequent presentation of a novel social stimulus (24 h). During the latter, a novel social odor (i.e., bedding soaked in a scent of an unfamiliar mouse of the same strain, sex, and age) and neutral olfactory cue (i.e., clean bedding) were put behind the perforated partitions of the opposing Eco-HAB^®^ chambers for the animals to voluntarily explore. Data were continuously recorded throughout the experiment and then evaluated. We analyzed activity, defined as the number of visits to all four Eco-HAB^®^ compartments, calculated in 12-h time bins, relative to the alternating dark and light phases of the light/dark cycle. To assess the exploration of a novel environment, we evaluated the animals’ activity during the first 12 h upon introduction of the cohort to the Eco-HAB^®^ chambers. We also analyzed in-cohort sociability*,* reflecting the tendency to voluntarily spend time with conspecifics, during the second and third dark phase of adaptation (i.e., during the period when the social structure of the tested cohort stabilized). As previously described by [21], in-cohort sociability was calculated as the total time that each pair of mice spent together minus the time that the animals would spent jointly because of their individual preferences for occupying certain spaces within the Eco-HAB^®^ chamber. We also evaluated approach to social odor, which was calculated as an increase in the proportion of time that each animal spent in the compartment with a novel social odor to the time spent in the compartment with a neutral-olfactory cue relative to the respective period from the preceding adaptation phase. For detailed information about the analyses of these measures, see [21].

### DiI staining

DiI staining was performed as described [23]. Dendritic spines from adult (P60) WT and KO mice were analyzed.

### Primary neuronal cell cultures

Dissociated primary hippocampal cultures were prepared from either P0 Wistar rats or P0 *Srf*^*f/f*^ transgenic mice as described previously [26] and plated at a density of 120,000 cells per 18-mm-diameter coverslip.

### DNA constructs and transfection

The pRNAT-H1.1/Shuttle-shSRF and pRNAT-H1.1/Shuttle-shCTR plasmids, both of which drive green fluorescent protein (GFP) expression, were transfected using Lipofectamine 2000 (Invitrogen, #11668019) on DIV6-7 according to the manufacturer’s protocol. The plasmids were provided by Dr. B. Paul Herring (Department of Cellular and Integrative Physiology, Indiana University School of Medicine, Indianapolis, Indiana, USA). For mouse cultures, CaMKIIα-GFP and CaMKIIα-Cre plasmids were transfected using Lipofectamine 2000. Dendritic spine morphology analyses were performed on DIV18-21. In the biochemical experiments that involved the transduction of adeno-associated virus (AAV1/2, isotype 1 and 2; AAV-shCTR, AAV-shSRF, AAV-CaMKIIα-mCherry, AAV-CaMKIIα-Cre), hippocampal neurons were transduced on DIV5-6 and collected for protein or RNA analysis on DIV18-21. The electroporation of freshly dissociated newborn hippocampal neurons was conducted using rat neuron nucleofection reagents (Amaxa, Lonza, Germany) with the 5×SRE-Luc reporter plasmid (Stratagene) and EF1αLacZ (β-galactosidase [β-Gal]), both of which were described previously [27].

### Luciferase and β-galactosidase reporter gene assays

Luciferase and β-Gal activity were evaluated in neuronal cell lysates using commercial assay kits (Promega, #E2000, #1500). Luminescence recordings were performed using an Infinite M200 microplate reader with an injector system (Tecan). To measure β-Gal activity, absorbance at 420 nm was read with a Sunrise 96-well plate reader (Tecan). Transcriptional activity was determined as luciferase activity normalized to β-Gal activity and compared with SRF-driven transcription on DIV3.

### Immunostaining and confocal microscopy

Hippocampal cultures were fixed and immunostained with mouse anti-GFP antibody (Millipore Cat# MAB3580), anti-Bassoon antibody (SySy, #141 003), anti-PSD-95 antibody (Millipore, #MABN68), or anti-SRF antibody (Santa Cruz, # sc-13029, #sc-335), which were followed by Fluor-488-, Alexa Fluor-546-, or Alexa Fluor-555-conjugated secondary antibody (Invitrogen, #A 11008; #A 21202, #A 10040, #A 32727). The analysis of the average fluorescence intensity of SRF was performed using ImageJ software (NIH). The background-corrected immunostaining intensity in GFP-positive, transfected cells was normalized to the intensity of non-transfected adjacent cells. For dendritic spine analysis, GFP-positive pyramidal neurons were examined under a confocal microscope (Leica TCS SP8) that was equipped with an HC PL APO CS2 63x/1.40 oil immersion objective using the 488-nm line of an argon laser. The pixel resolution was 2048 × 2048, and the resulting pixel size was 0.07 μm. The sum of *Z*-stacks (maximum intensity projections) of secondary dendrites was analyzed. For the analysis of synaptic markers, GFP-positive pyramidal neurons were acquired on a Zeiss LSM 800 Airyscan confocal microscope with a PL APO 63x/1.4 oil immersion objective using 488/561/640 nm diode lasers with sequential acquisition settings of 1024 × 1024 resolution, 2 × optical zoom, and 0.05 × 0.05 μm pixel size. The settings were kept the same for all scans.

### Dendritic spine morphology analysis

Confocal images of selected dendritic spine segments were semi-automatically analyzed using SpineMagick! Software (international patent no. WO/2013/021001) as previously described [28]. Spine shapes were divided into clusters and then sorted into two groups, “filopodia and long” and “mushroom and stubby,” using custom scripts [29].

### Surface receptor staining of living hippocampal neurons

Hippocampal cultures from P0 rats were transfected with pRNAT-H1.1/Shuttle-shSRF or pRNAT-H1.1/Shuttle-shCTR plasmid as described above. Levels of GluA1- or GluA2-containing AMPARs were assessed on DIV18. To label surface AMPARs, anti-GluA1 or anti-GluA2 antibody (Sigma, #ABN241 and #MAB397, respectively), diluted to a final concentration of 10 μg/ml, were added to the neuronal cultures and incubated at 37 °C for 15 min. Unbound antibodies were quickly washed with ice-cold Minimum Essential Medium MEM) and then the cells were placed in fixation medium (4% formaldehyde/4% sucrose/phosphate buffer [PB]) for 7 min. Alexa Fluor-555 or Alexa Fluor-546-conjugated secondary antibodies (1:100; Invitrogen, #A 10040, #A 32727) for appropriate species were diluted in GDB buffer (0.1% bovine serum albumin, 17 mM PB, and 0.45 M NaCl) without Triton and incubated at room temperature for 1 h. After washing, coverslips were mounted with ProLong Gold (Invitrogen, #P10144). Transfected, GFP-positive pyramidal neurons were examined under a Leica TCS SP8 confocal microscope according to the previous experiments.

### Image analysis of SRF immunofluorescence

Z-stack-based Maximum Intensity projections (16 bit) of coronal brain sections stained with Hoechst 33342 and SRF-antibody were processed using ImageJ software. The Hoechst 33342 signal was thresholded and used to make a selection (ROI) for hippocampal fields (CA1, DG). The created ROIs were then restored over channel with SRF-immunofluorescence to analyze “*Integrated density*” representing the sum of the signal values of the pixels within selection. The obtained data were normalized to control.

### Image analysis of synaptic markers and surface receptor staining

*Z*-stack-based Maximum Intensity projections (16 bit) of secondary and tertiary dendrites of GFP-positive neurons were processed using ImageJ software. Based on the GFP signal, the mask of transfected neurons was created and applied to other fluorescent channels. For each neuron, at least three dendritic segments were selected for further analysis. The signal from the masked segments of each channel was thresholded, based on a subjective evaluation of real puncta “clusters” compared with background noise. The same threshold was kept for each experiment. To separate overlapping objects, the “*Watershed separation*” function was implemented, and then the “*Analyze Particles*” option was applied. The pixel area size was set based on experimenter picture evaluation to exclude anything that was not an object of interest in the image. The results were then added to ImageJ “*Manager*” and then restored over each fluorescent channel to analyze the area, fluorescence intensity, and density of particles. The average value of each parameter was created per one analyzed cell.

To analyze the colocalization of Bassoon/PSD-95 within dendritic spines, the Pearson correlation coefficient was used. The colocalization of two immunofluorescent channels was calculated individually in the regions restricted to the interior of precisely marked dendritic spine contours. Thus, it measures a correlation between the fluorescence intensities of two fluorophores and reflects their molecular interaction. The analysis was performed using the SpineMagick! program. The value of the coefficient varies from -1 to 1. Values closer to 1 indicate a higher degree of colocalization. Values closer to 0 represent a lack of correlation between the signals.

### Electrophysiology

The patch-clamp technique was used to measure miniature excitatory postsynaptic currents (mEPSCs). Hippocampal neurons were grown on glass coverslips and incubated in artificial cerebrospinal fluid solution (ACSF; 119 mM NaCl, 2.5 mM KCl, 1.3 mM MgCl_2_, 1 mM NaH_2_PO_4_, 26 mM NaHCO_3_, 20 mM D-glucose, and 2.5 mM CaCl_2_, saturated with carbogen) supplemented with 100 μM picrotoxin (Abcam, #U 93631) and 0.5 μM tetrodotoxin (Tocris, # 078) and heated to 31 °C. GFP-positive pyramidal cells were identified visually and patched with borosilicate glass capillaries (4–6 MΩ resistance) that were filled with Cs-based internal solution (130 mM Cs-gluconate, 20 mM HEPES, 3 mM TEA-Cl, 0.4 mM EGTA, 4 mM Na_2_ATP, 0.3 mM NaGTP, and 4 mM QX-314Cl, pH 7.0–7.1; osmolarity: 290–295 mOsm). To measure mEPSCs, approximately 20-min-long voltage-clamp recordings were collected. Miniature events were analyzed using MiniAnalysis software (Synaptosoft) with an amplitude detection threshold set to 7 pA. All mini-events that were automatically detected by the software were visually verified by the experimenter.

### BS^3^ staining

To assess surface protein levels, the crosslinking with bis(sulfosuccinimidyl)suberate (BS^3^; ThermoFisher, # 21580) protocol was applied according to [30]. Briefly, AAV-infected cultures, after three washes with fresh maintenance medium, were incubated with 2 mM BS^3^ for 10 min with agitation at 37 °C. Crosslinking was terminated by quenching the reaction with 100 mM glycine (10 min, 4 °C). Protein extracts were then probed using standard Western blot procedures.

### Western blot analysis

Twenty micrograms of protein extracts collected from in vitro mice or rat cultures were run on polyacrylamide gels (BioRad, #4569033) under reducing conditions [23]. The standard Western blot procedure was performed using anti-GluA1 (Sigma, #ABN241), anti-GluA2 (Sigma, #MAB397), and anti-SRF (Santa Cruz, #sc-13029) antibodies. To monitor equal total protein levels, the blots were re-probed with α-tubulin (Sigma, # T9026) antibodies. For signal detection, the chemiluminescent method was used. To quantify individual bands, a scan of X-ray films was analyzed by densitometry using GeneTools software (Syngene).

### RNA preparation and quantitative real-time polymerase chain reaction

Total RNA was isolated from rat or mouse hippocampal cultures using the RNeasy Mini Kit (Qiagen, #74104). RNA was reverse transcribed with SuperScript IV Reverse Transcriptase (Invitrogen #18090050) according to the manufacturer’s instructions. cDNA was amplified with TaqMan probes (ThermoFisher) that were specific for mouse or rat. Quantitative real-time polymerase chain reaction (PCR) was performed using Fast TaqMan Master Mix (Applied Biosystems, #44456) with an Applied Biosystems 7900HT Fast Real-Time PCR System. Fold changes in expression were determined using the ∆∆CT relative quantification method. The values were normalized to relative amounts of Gapdh.

### Statistical analyses

To compare the distributions of datasets, the Shapiro–Wilk normality test was performed. Unpaired Student’s *t*-test or the Mann–Whitney test (nonparametric) was used to test differences between two groups. When required, one- or two-way analysis of variance (ANOVA) was performed, followed by Sidak’s multiple-comparison post hoc test. To compare cumulative distributions of data, the Kolmogorov–Smirnov test was used. The number of neurons and independent cultures or animals that were used for the analyses are reported in the appropriate Figure legends. Values of *p* < 0.05 were considered statistically significant. The results were analyzed using GraphPad Prism software.

## Results

### SRF regulates distinct aspects of social behavior and dendritic spine maturation in the hippocampus

**Fig. 1:**
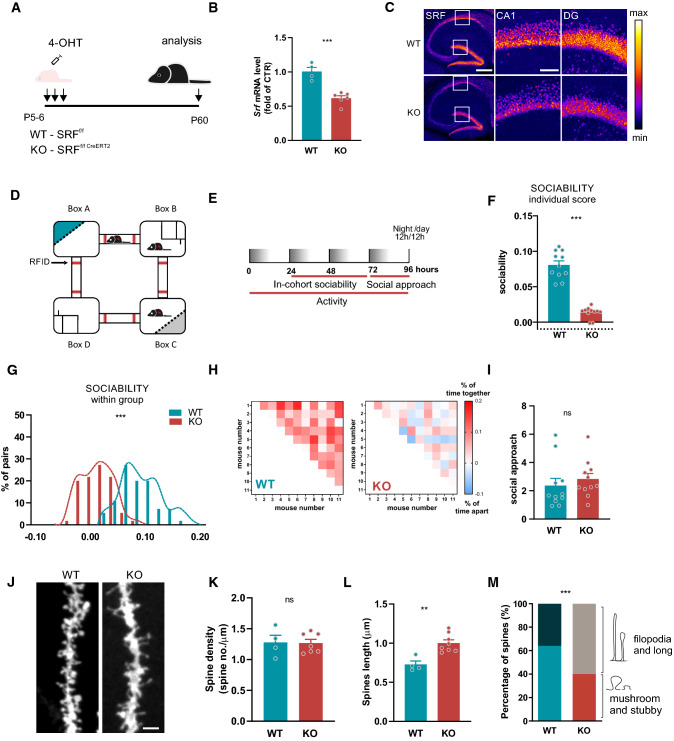
SRF KO male mice exhibit a decrease in sociability despite normal responses to novel social stimuli together with dendritic spine maturation impairments. **a** Diagram of the experimental design of the early postnatal deletion of SRF in excitatory neurons. **b** Analysis of Srf knock-out efficiency by 4-OHT injections in adult mice hippocampus, WT*n*_*mice*_ = 4; KO*n*_*mice*_ = 6. **c** Conditional deletion of SRF during development analyzed in the adult hippocampus. Immunofluorescent staining of SRF in CTR and SRF KO animals. SRF elimination can be observed in the dentate gyrus (DG), and CA1 subfield of the hippocampus. Scale bar = 400 µm. White squares correspond to enlarged images of CA1 and DG, scale bar = 100 µm. The corresponding color histogram depicts the SRF fluorescence intensity (*min—max*) in a false-color scheme (‘*Fire* LUT’). **d** Eco-HAB^®^ scheme. The cage consists of four housing compartments (boxes) connected by tube-shaped passages equipped with RFID antennas that track the individual behavior of each mouse. Boxes B and D contain food and water, and boxes A and C contain impassable, perforated partitions (dashed lines), behind which social (blue) and non-social (gray) cues are presented. **e** Experimental timeline applied to test mice sociability (**f**,**g**,**h**) and social approach (**i**). **h** Raw data from (**f**) and (**g**), represented as a matrix in which squares illustrate the time voluntarily spent together by each pair of mice within the cohort. The intensity of colors reflects the strength of the relationship in accordance with the presented scale. WT*n*_*mice*_ = 11; KO*n*_*mice*_ = 11. **j** Example images of DiI-stained dendrites with dendritic spines from the CA1 area in wildtype (WT) and SRF KO animals together with analysis of spine density (**k**), length (**l**) and percentage of protrusions that clustered into two morphological categories (**m**). Scale bar = 2 µm. WT*n*_*mic*e_ = 4; KO*n*_*mice*_ = 7. Student’s *t*-test used in (**b**,**k**,**l**), Mann–Whitney test in (**c**,**f**), Kolmogorov–Smirnov test in (**d**) and *χ*^*2*^ in (**m**). Data in (**m**) *χ*^*2*^ = 11.54, *df* = 1. Data as means ± SEM. ^*ns*^*p* > 0.05, **p < 0.01, ***p < 0.001

In the current study, we have developed a new transgenic mouse model with specific, neuronal SRF deletion circumventing early postnatal death. To delete SRF exclusively in the forebrain excitatory neurons, we used conditional SRF mutants (*SRF*^*f/f*^) that were crossed with a tamoxifen-inducible CRE recombinase line under the CaMKIIα promoter (CaMKCreER^T2^). Then, starting on P5-6, *Srf*^*f/f*^ and *Srf* ^*f/fCaMKCreERT2*^ pups were injected with three doses of 4-Hydroxytamoxifen (4-OHT), every other day, to stimulate the translocation of Cre recombinase to the nucleus and *SRF* deletion (Fig. 1a). Resulting young adult *Srf*^*f/f*^ (wild type, WT) and *Srf* ^*f/f;CaMKCreERT2*^ (SRF gene knock-out mice carried a single copy of Cre recombinase, KO) were used for the experiments. We have confirmed that endogenous CaMKIIα expression starts already at P2 in the mouse hippocampus (Fig. S1a), well before neuronal maturation is completed. Next, we have verified Cre-recombinase expression at P9 in CaMKCreER^T2^ mice by injecting animals with three doses of 4-OHT, every other day (Fig. S1b). 24 h after the last injection, animals were sacrificed, perfused, and their brains stained with an anti-Cre antibody. We have found nuclear localization of Cre protein in the cerebral cortex and the hippocampus (Fig. S1b). Additionally, we used a double transgenic reporter mice line Ai14 (*loxP*-flanked STOP tdTomato) CaMKCreER^T2^ and confirmed 4-OHT-induced recombination in the mouse brain 24 h after the last from three injections (Fig. S1c). Then, the efficiency of induction of SRF knock-out was assessed by qRT-PCR and immunostaining. *Srf*^*f/f;CaMKCreERT2*^ (KO) had significantly decreased SRF mRNA levels in the young, P12-14 (Fig. S1d, *p* = 0.0005) and adult hippocampus, P60 (Fig. 1b) as well as reduced immunofluorescence staining against SRF at the slices from young, P19 (Fig. S1e), and the adult brain (Fig. 1c). KO mice exhibited a significant decrease in SRF immunofluorescence in CA1 and DG at 10 days after the last from three 4-OHT injections (Fig. S1f, CA1 *p* = 0.0196; DG *p* = 0.0318).

Having a validated model of early postnatal SRF deletion, we have carried out behavioral tests, applying long-term monitoring of mouse behavior focusing on distinct aspects of animals social behavior, impairments of which are core symptoms of the autistic phenotype that is frequently observed in animal studies of the disorder [20]. We used Eco-HAB^®^, an RFID (radio-frequency-based identification)-based system for the precise, automated assessment of individual, voluntary social behavior in group-housed mice under semi-natural conditions [21]. Eco-HAB^®^ consists of four individual compartments connected by tube-shaped corridors. Two of them provide shelter and access to food and water. The other two chambers contain transparent, impassable perforated partition behind which an olfactory stimulus may be presented (Fig. 1d). The behavior of housed together cohorts of male WT and SRF KO mice was continuously recorded for 96 h (Fig. 1e). Social behavior was measured after 24 h of habituation to the novel Eco-HAB^®^ environment when the group’s social structure stabilized. We found that SRF KO animals exhibited severe impairments in the pattern of interactions with familiar mice (in-cohort sociability), measured as the time voluntarily spent together with other animals within the group (Fig. 1f–h). SRF KO mice were less willing to spend time together compared with WT animals (Fig. 1f; *p* < 0.0001, g; *p* < 0.0001). Despite this, social approach, i.e., an interest in novel social stimuli, measured as a proportion of time spent exploring social *vs*. non-social scents that were presented behind the perforated partitions of the opposing Eco-HAB^®^ cages, was robust and had a similar intensity in WT and SRF KO male mice (Fig. 1i; *p* = 0.1932). The observed social behavior in SRF KO mice is highly specific and not confounded by other factors such as anxiety (Fig. S2a; *p* = 0.9588), disrupted activity (Fig. S2b; *p* = 0.18745, c; *p* = 0.3101), inability to follow conspecifics and thus respond to social cues (Fig. S2d; *p* = 0.4309) or impaired ability to locate stimuli (Fig. S2e; *p* = 0.4619).

As currently recommended, we tested the SRF KO social phenotype in both sexes [31, 32]. Similarly to males, SRF KO females exhibited a significant decrease in in-cohort sociability (Fig. S3a; *p* = 0.0013, b; *p* < 0.0001, c), albeit to a lesser extent. Opposed to males, females’ interest in novel social stimuli (social approach) was increased (Fig. S3d; *p* = 0.0464). Altogether, an early postnatal SRF deletion impairs mice social behavior. The observed behavioral phenotype share some similar traits commonly observed in autism spectrum disorder.

To establish if behavioral consequences of early postnatal deletions of SRF in hippocampal excitatory neurons have underlying structural changes in the brain, we measured the density and morphology of dendritic spines in the *stratum radiatum* of the hippocampal CA1 region in adult animals (Fig. 1j). The hippocampus has been recognized as of particular interest when studying different aspects of social interactions. The analysis of spine density showed no significant changes in the number of spines (Fig. 1k; *p* = 0.9248), however, we observed a substantial increase in dendritic spine length in SRF KO animals (Fig. 1l; *p* = 0.0021). To investigate how SRF deletion affects dendritic spine shape, we clustered spines into two morphological categories (“filopodia and long spines” and “mushroom and stubby") (Fig. 1m). At an age when the majority of excitatory postsynaptic structures exhibit a mushroom shape (6–8-week-old mice), we observed a significant shift in the spine morphology of SRF-KO neurons to filopodia-like, immature protrusions compared with control cells in WT animals. SRF KO mice exhibited a significant 24 percentage points increase in the occurrence of filopodia and long spines and a decrease in the population of mushroom spines in the CA1 compared with wildtype animals (Fig. 1m; *p* = 0.0021). Of note, we did not observe any gross changes in hippocampal lamination or axonal pathfinding in SRF KO mice (Fig. S4a, b).

### Depletion of SRF reduces the number of mature spines in vitro

**Fig. 2:**
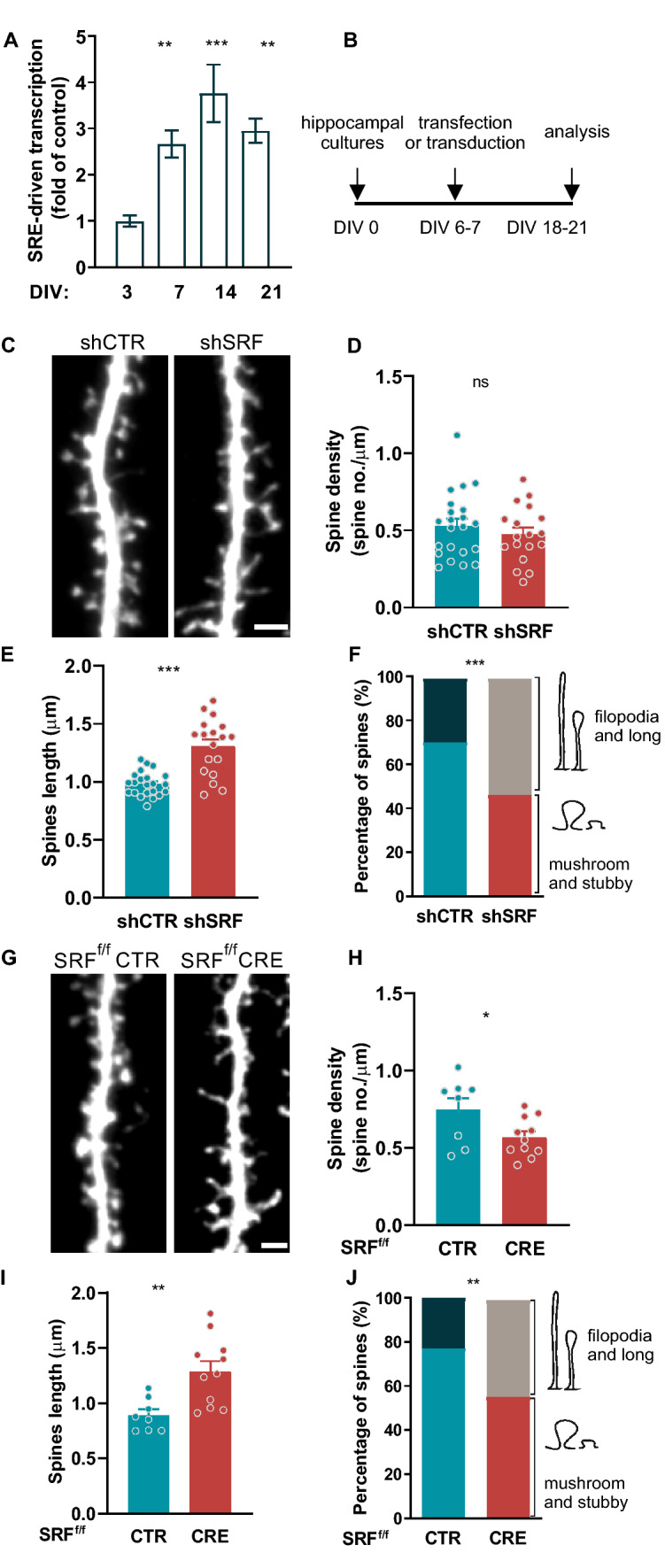
SRF regulates dendritic spine maturation in vitro. **a** SRF-driven transcription in rat hippocampal cultures on DIV3, 7, 14, 21 reflected by luciferase/β-Gal activity ratio. *n*_*samples*_ > 8. **b** Diagram of the experimental design. **c**–**f** Example images (**c**) together with the analysis of spine density (**d**), length (**e**), and percentage of protrusions clustered into two morphological categories (**f**) in rat hippocampal neurons transfected with shCTR or shSRF plasmid. shCTR*n*_*cells*_ = 22; shSRF*n*_*cells*_ = 18. **g**–**j** Example images (**g**) together with the analysis of spine density (**h**), length (**i**), and percentage of protrusions clustered into two morphological categories (**j**) in mouse hippocampal neurons transfected with a control vector (CTR) or CRE. AAV-CTR*n*_*cells*_ = 8; AAV-CRE*n*_*cells*_ = 11. One-way ANOVA F_3,31_ = 11.67; *p* < 0.0001 with Sidak’s post hoc test used in (**a**), Student’s *t*- test in (**c**–**e**; **g**–**i**) and *χ*^*2*^ in (**f**, **j**). Data in (**f**) *χ*^*2*^ test = 11.99, *df* = 1, (**j**) *χ*^*2*^ test = 10.24 *df* = 1. Data as means ± SEM. ^*ns*^*p* > 0.05, *p < 0.05, **p < 0.01, ***p < 0.001. All data obtained from at least three independent cultures. Scale bars (**c**,**g**) = 2 µm

Our data indicate that SRF deletion modulates animals behavior, and that these changes are associated with immaturity of dendritic spines in vivo. To understand the molecular mechanism underlying this phenomenon, and whether SRF-driven transcription in neurons might be developmentally regulated, we analyzed SRF transcriptional activity in vitro. On DIV0, rat hippocampal neurons were electroporated using Amaxa with a 5×SRE reporter construct that contained five CArG boxes. SRF-driven transcription was analyzed on DIV3, 7, 14, and 21. An increase in transcription levels was observed on DIV7—21, with the highest level detected on DIV14 (Fig. 2a; DIV3 *vs*. DIV7; *p* < 0.0001, DIV3 *vs*. DIV14; *p* = 0.0011, DIV3 *vs*. DIV21). These results suggest that SRF-dependent transcription was developmentally regulated.

To further evaluate the impact of SRF on synaptogenesis and dendritic spine maturation, we used the Lipofectamine-based transfection method to visualize only sparsely stained neurons and to precisely analyze spine morphology (Fig. 2b). We assessed the effects of SRF deficiency on the density and morphology of dendritic spines (Fig. 2c–f). Neurons were transfected with either shRNA (shSRF) or scrambled shRNA (shCTR) plasmids at DIV6-7 and fixed on DIV18-21. The density analysis showed no significant changes in the number of dendritic protrusions on DIV21 between shCTR- and shSRF-transfected neurons (Fig. 2c), suggesting that SRF is not required for dendritic spine formation (Fig. 2d; *p* = 0.39). However, on DIV18-21, we observed a significant increase in dendritic spine length in SRF-depleted rat neurons (Fig. 2e; *p* < 0.001). The efficiency of the shRNA-induced SRF knock-down was confirmed in rat hippocampal cultures in vitro by immunofluorescence (Fig. S5a,b). To further investigate how the lack of SRF affects dendritic spine shape, we again clustered spines into two distinct morphological categories: “filopodia and long spines” and “mushroom and stubby spines “. Although SRF deficiency did not affect the number of spines, the early developmental deletion of SRF significantly increased by 24 percentage points the proportion of filopodia and long spines, with a significant decrease in the population of mushroom and stubby spines (Fig. 2f) compared with shCTR neurons. To exclude the possibility that shSRF may act nonspecifically, we performed an independent analysis using mouse neurons isolated from the hippocampus of *Srf*^*f/f*^ mice that were transfected with a plasmid-encoding Cre recombinase under the CamKIIα promoter on DIV6-7 (Fig. 2g). Cre-dependent SRF deletion was confirmed in *Srf* ^*f/f*^ mouse hippocampal neurons (Fig. S5c, d). Similar to the shRNA results, neurons that expressed Cre recombinase also exhibited an increased spine length (Fig. 2i; *p* = 0.0039) together with a lower percentage of mature dendritic spines and an increase in immature protrusions (Fig. 2j;  21 percentage points of filopodia and long spines), independently confirming that SRF facilitates structural spine maturation. In this experiment, we have observed a significant difference in dendritic spine density between analyzed groups (Fig. 2h; *p* = 0.0997).

### SRF regulates excitatory synaptic transmission and the number of functional synapses

**Fig. 3:**
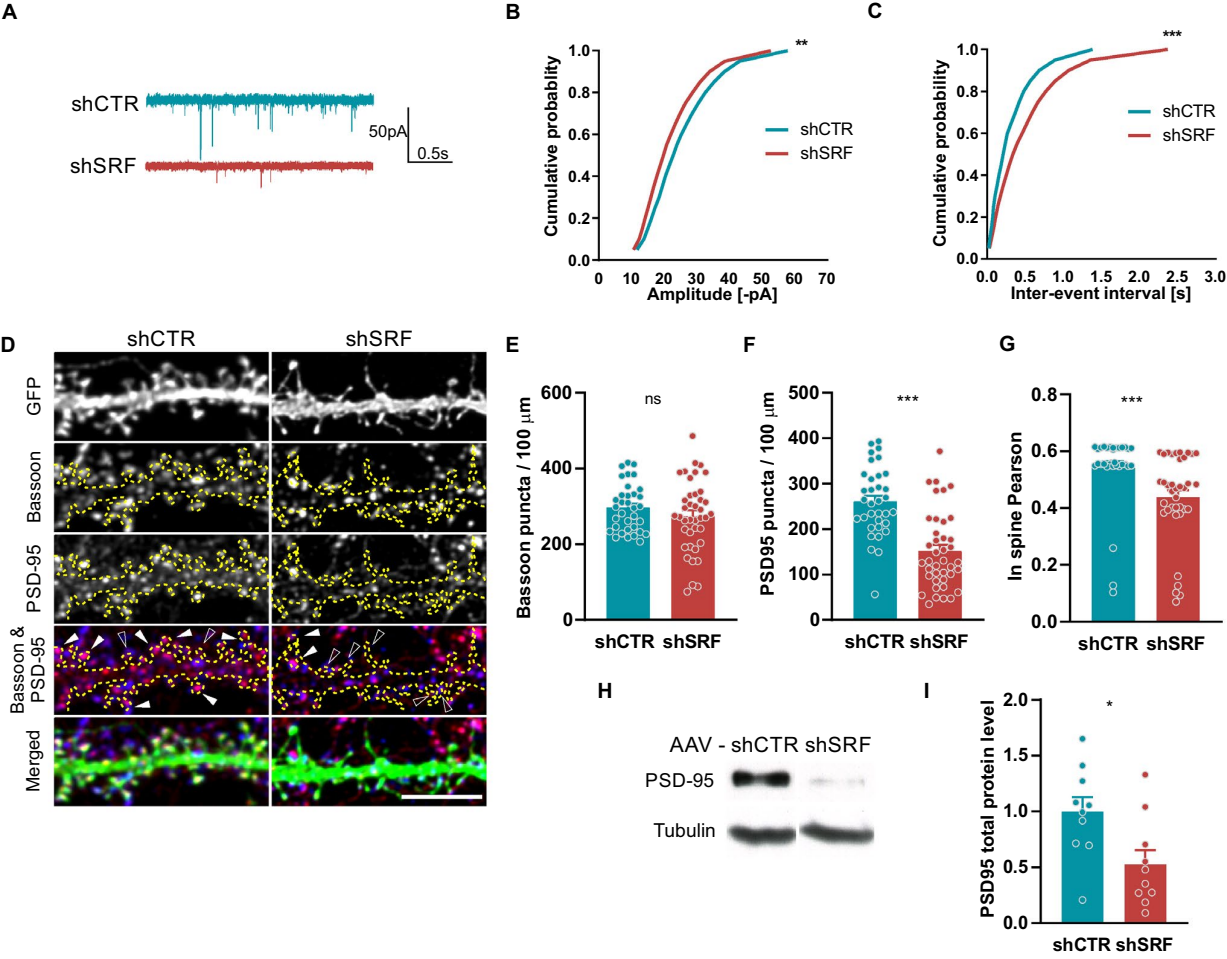
SRF knock-down impairs AMPAR transmission and number of functional synapses in hippocampal cultures. **a–c** Sample traces of mEPSCs (**a**), together with cumulative probability plots of mEPSC amplitude (**b**) and frequency (**c**) in shCTR and shSRF-transfected neurons. shCTR*n*_*cells*_ = 8; shSRF*n*_*cells*_ = 10. **d** Example images of dendrites of shCTR- and shSRF-transfected neurons that were double-immunostained with anti-Bassoon (blue) and anti-PSD-95 (red) antibodies. Dendrites are outlined. In the red/blue color scheme, the colocalization of both synaptic markers is shown as purple immunofluorescence. White arrowheads indicate an overlap of both markers within the spine. Empty arrowheads indicate Bassoon-positive/PSD-95-negative dendritic spines. **e**, **f** Bassoon and PSD-95 puncta density within dendritic segments. shCTR*n*_*cells*_ = 34; shSRF*n*_*cells*_ = 40. **g** Overlap of Bassoon and PSD-95 puncta in spines expressed as Pearson’s coefficient. shCTR*n*_*cells*_ = 32; shSRF*n*_*cells*_ = 37. **h** Western blots of PSD-95 total protein expression in extracts from AAV-shCTR- and AAV-shSRF-transduced rat hippocampal neurons. **i** Total PSD-95 from (**h**). The data are presented as a fold change relative to control. shCTR*n*_*samples*_ = 10; shSRF*n*_*samples*_ = 10. Kolmogorov–Smirnov test used in (**b**, **c**), Mann–Whitney test in (**e**–**g**) and Student’s *t*-test in (**i**). Only Bassoon puncta density data (**e**) passed the normality test, ^*ns*^*p* = 0.4105 (Student’s *t*-test). Data as means ± SEM. ^*ns*^*p* > 0.05, *p < 0.05, **p < 0.01, ***p < 0.001. Western blot data from five independent cultures. Scale bar = 5 µm

Assuming that SRF activity is crucial for the structural maturation of dendritic spines, we tested whether SRF deletion leads to functional alterations of excitatory synaptic transmission. Hippocampal neurons were transfected on DIV6 with either shSRF or shCTR. On DIV21, AMPAR-mediated miniature excitatory postsynaptic currents (AMPAR-mEPSCs) were recorded from GFP-expressing neurons (Fig. 3a–c).

Comparisons of the cumulative probability of cellular responses between shCTR- and shSRF-transfected neurons demonstrated a significant decrease in mEPSC amplitude (Fig. 3b; *p* = 0.0080) and frequency (Fig. 3c; *p* = 0.0002), indicating impairments in excitatory synaptic transmission. The increase in the time of inter-events intervals of mEPSCs in SRF-depleted cells might indicate a lower number of mature synapses.

The alterations in spine morphology were associated with decreases in the amplitude and frequency of mEPSCs in SRF-depleted neurons, and no differences in spine density were detected between the analyzed groups. Thus, the changes in synaptic transmission may have resulted from a postsynaptic defect. To test whether a lower number of functional synapses underlies reduced mEPSC frequency, we quantified markers of the excitatory synapses as a morphological readout of neuronal connectivity. On DIV21, we measured the number of puncta that were stained with presynaptic (Bassoon) and postsynaptic (PSD-95) proteins in hippocampal neurons that were transfected with either shCTR or shSRF (Fig. 3d). The quantification of Bassoon-positive puncta density at selected dendritic segments revealed no significant changes between groups (Fig. 3e; *p* = 0.4105). Simultaneously, further analysis of the same dendrites revealed that shSRF-transfected neurons exhibited a decrease in PSD-95 puncta density (Fig. 3f; *p* < 0.0001) compared with control cells. Moreover, the immunofluorescence staining of neurons revealed a decrease in the synaptic overlap of Bassoon and PSD-95 proteins in shSRF-transfected cells, reflected by a lower Pearson’s correlation coefficient (Fig. 3g; *p* < 0.0001). To check a possible source of lower number of PSD-95 puncta in SRF-depleted neurons we examined total PSD-95 protein in shCTR and shSRF transfected cells (Fig. 3h, i). SRF knockdown decreased the total level of PSD-95 protein (Fig. 3i; *p* = 0.017). Altogether, these results suggest that the number of functional synapses decreased after silencing SRF expression during neuronal development in vitro.

### SRF knock-down decreases surface levels of AMPARs

**Fig. 4:**
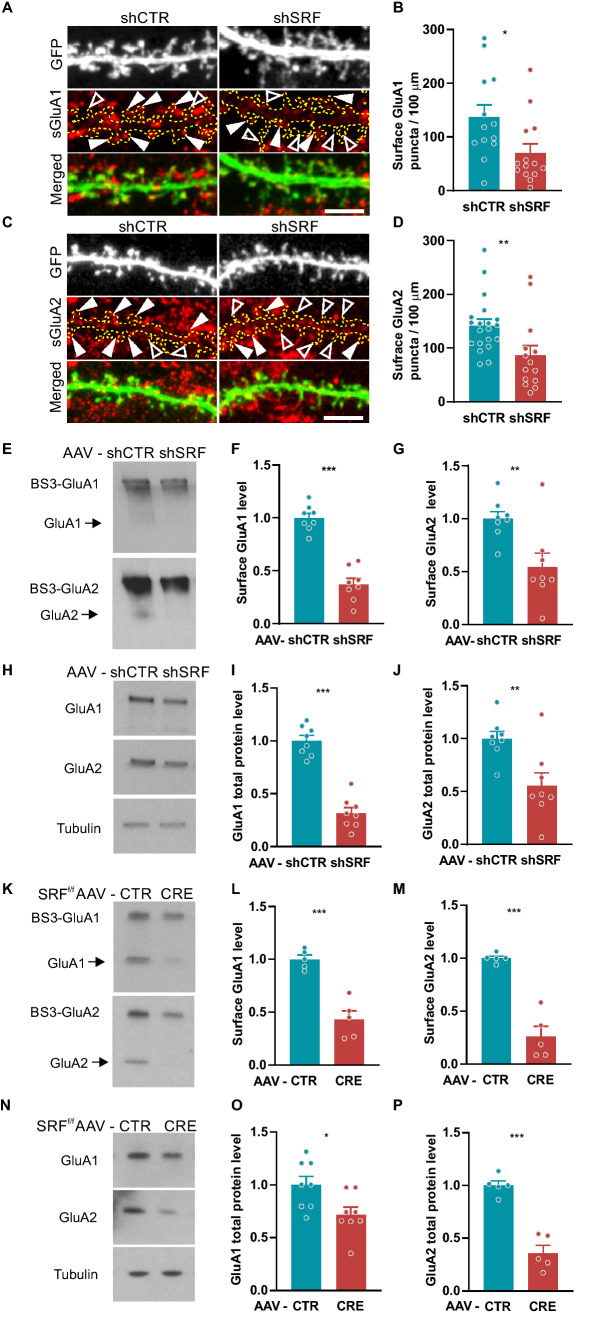
SRF depletion reduces surface GluA1 and GluA2 AMPAR subunit levels in rat hippocampal neurons. **a** Example images of dendrites of shCTR- or shSRF-transfected neurons surface-immunostained by anti-GluA1 antibody. Dendrites are outlined. White arrowheads indicate examples of GluA1-positive spines. Empty arrowheads indicate examples of spines that lack GluA1 expression. **b** GluA1 puncta density localized within the dendritic segment. shCTR*n*_*cells*_ = 13; shSRF*n*_*cells*_ = 14. **c** Example images of dendrites of shCTR- or shSRF-transfected neurons surface-immunostained by anti-GluA2 antibody. Dendrites are outlined. Examples of GluA2-positive spines are indicated by white arrowheads. Empty arrowheads indicate examples of spines that lack GluA2 expression. **d** GluA2 puncta density localized at dendritic segments. shCTR*n*_*cells*_ = 13; shSRF*n*_*cells*_ = 14. **e** Western blots of surface proteins in extracts from AAV-shCTR- and AAV-shSRF-transduced rat hippocampal neurons. Surface GluA1 and GluA2 were BS^3^ crosslinked before protein extraction. Arrows indicate the intracellular pool of receptors. **f**, **g** Surface GluA1 and GluA2 protein levels from (**e**). shCTR*n*_*samples*_ = 8; shSRF*n*_*samples*_ = 8. **h** Western blot of GluA1 and GluA2 total protein expression in AAV-shCTR- and AAV-shSRF-transduced rat hippocampal neurons. **i**, **j** Total GluA1 and GluA2 protein levels from (**h**). shCTR*n*_*samples*_ = 8; shSRF*n*_*samples*_ = 8. **k** Western blots of BS^3^ crosslinked surface proteins in extracts from CTR- and CRE-AAV- transduced *Srf*^*f/f*^ mouse hippocampal neurons. Arrows indicate the intracellular poll of receptors. **l**, **m** Surface GluA1 and GluA2 protein levels from (**k**). **n** Western blot of GluA1 and GluA2 total protein expression in AAV-CTR- and AAV-CRE-transduced *Srf*^*f/f*^ mouse hippocampal neurons. **o**, **p** Total GluA1 and GluA2 protein levels from (**n**). Data are presented as a fold change relative to control. Mann–Whitney test used in (**b**, **d**) and Student’s *t*-test in (**f**, **g**, **i**, **j**, **l**, **m**, **o**, **p**). Data as means ± SEM. *p < 0.05, **p < 0.01, ***p < 0.001. Western blots data from four independent cultures. Scale bars (**a**,**c**) = 5 µm

To further investigate the functional significance of the shift in spine morphology toward a more immature phenotype, and to understand the underlying molecular pathway, we analyzed the impact of SRF deletion on surface AMPAR expression. To measure the level of surface-exposed GluA1 and GluA2 subunits, we used a live-staining method with antibodies that were directed against the extracellular N-terminal domains of GluA1 or GluA2 subtypes. The labeling procedure was performed in hippocampal neurons that were transfected with shCTR or shSRF (Fig. 4a). SRF depletion significantly reduced the number of surface GluA1 (Fig. 4b; *p* = 0.0145) and GluA2 (Fig. 4d; *p* = 0.0017) puncta, suggesting a decrease in postsynaptic AMPAR levels, which was consistent with the reduction of mEPSC amplitude in shSRF-transfected neurons (Fig. 4b). Altogether, these data demonstrate that SRF regulates AMPAR-dependent excitatory synaptic transmission.

To validate the results that showed the decrease in surface GluA1 and GluA2 levels in shSRF-transfected neurons, we performed a biochemical analysis of SRF-depleted rat and mice hippocampal cultures (Fig. S6a,c) and confirmed the efficiency of the AAV-induced knock-down of SRF using Western blot (Fig. S6b,d). AAV-shSRF and AAV-Cre constructs effectively diminish SRF expression in neurons in vitro. Next, to stain surface receptors, we used the membrane-impermeant chemical crosslinking reagent *BS*^*3*^*,* which selectively distinguishes cell surface proteins [30, 33]. The immunoblot analysis of AMPARs in crosslinked neurons (Fig. 4e) revealed a decrease in surface GluA1 and GluA2 subunit levels in cultures that were transduced with AAV-shSRF particles compared with AAV-shCTR (Fig. 4f; *p* < 0.0001, g; *p* = 0.0076). To test whether the decrease in surface GluA1 and GluA2 levels was attributable to the downregulation of total AMPAR protein levels, Western blot analysis was performed using independent extracts from the same biological experiment (Fig. 4h). Similar to the crosslinking experiments, cultured hippocampal neurons were transduced with AAV-shSRF or AAV-shCTR on DIV5, and total protein levels were analyzed on DIV21 (Fig. 4i; *p* < 0.0001, j; *p* = 0.0061). The total protein level of GluA1 and GluA2 also significantly decreased in SRF-depleted neurons compared with control cells.

To verify the results that were obtained by shRNA, we performed an independent analysis using mouse neurons that were isolated from the *Srf*^*f/f*^ mouse hippocampus that was transduced with AAV-Cre recombinase or AAV-CTR on DIV5-6. Similar to neurons that were transduced with AAV-shRNA, SRF deletion by CRE recombinase expression decreased both membrane-bound GluA1 and GluA2 (Fig. 4k, l; *p* = 0.0002, m; *p* < 0.0001) and total protein levels (Fig. 4n, o; *p* = 0.0202, p; *p* < 0.0001). The surface and total protein expression of GluA1 and GluA2 subunits were significantly reduced by SRF deletion in both models.

### Downregulation of *Gria1* and *Gria2* mRNA expression in SRF-depleted neurons

**Fig. 5:**
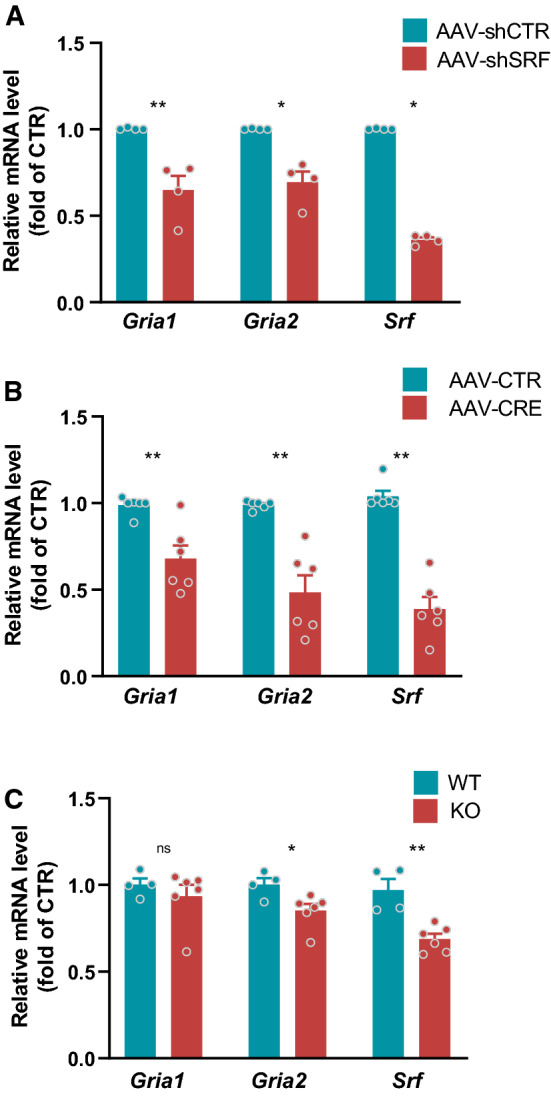
Developmental SRF deletion reduces *Gria1* and *Gria2* expression in hippocampal neurons in vitro and in vivo. **a** q-RT-PCR analysis of *Gria1*, *Gria2*, and *Srf* mRNA expression in rat neurons that were transduced with AAV-shCTR or AAV-shSRF. AAV-shCTR*n*_*cultures*_ = 4; AAV-shSRF*n*_*cultures*_ = 4*.* **b** *Gria1*, *Gria2*, and *Srf* mRNA quantification in AAV-CTR- and AAV-CRE-transduced *Srf*^*f/f*^ mouse neurons. AAV-CTR*n*_*cultures*_ = 6; AAV-CRE*n*_*cultures*_ = 6. **c** q-RT PCR analysis of *Gria1* and *Gria2* expression in WT and SRF KO mice. WT*n*_*mice*_ = 4; KO*n*_*mice*_ = 6. Mann–Whitney test used in (**a**,**b**), Student’s *t*-test in (**c**) Only *Gria2* data from mouse cultures in (**b**) passed the normality test, **p = 0.0005 (Student’s *t*-test). Data as means ± SEM. *p < 0.05, **p < 0.01

The molecular mechanisms by which SRF regulates synaptic plasticity are linked to its transcriptional activity and regulation of expression of several activity-induced genes [22]. To get better insight into the mechanism, we investigated whether SRF controls GluA1 (encoded by the *Gria1* gene) and GluA2 (encoded by the *Gria2* gene) gene expression during neuronal maturation, and conducted qRT-PCR. We confirmed that both mRNA and protein level of GluA1 and 2 increase over time in neurons in vitro (Fig. S7a-b), which correlates with the developmental increase of SRF-driven transcription level in culture (Fig. 2a). Then, we evaluated the expression of *Gria1* and *Gria2* mRNA in hippocampal neurons that were transduced with AAV-shSRF and AAV-shCTR on DIV5-6. Both mRNAs that encode AMPARs were downregulated in extracts from SRF-depleted rat hippocampal neurons on DIV21 (Fig. 5a; *p* = 0.0286 for *Gria1,2*). We also confirmed that AAV-shSRF significantly downregulated *Srf* mRNA levels (Fig. 5a).

Moreover, the results were validated by an independent analysis using *Srf*^*f/f*^ mouse neurons from the hippocampus that were transduced with AAV-Cre recombinase particles or AAV-CTR on DIV 5–6. Similar to the shRNA results, SRF deletion in mouse neurons decreased both *Gria1* and *Gria2* mRNA levels (Fig. 5b; *Gria1 p* = 0.0043, *Gria2 p* = 0.0022, *Srf p* = 0.0022). These results suggest that SRF regulates AMPAR expression at the transcriptional level.

To confirm the aforementioned results in vivo, we performed the qRT-PCR analysis of mRNA isolated from WT and KO animals to measure SRF-related changes in AMPAR expression (Fig. 5c,d). We found significantly diminished *Gria2* mRNA expression (Fig. 5d; *p* = 0.0292) and a slight, yet insignificant decrease mRNA level of *Gria1* (Fig. 5c; *p* = 0.4614). Similarly to the results obtained in vitro*,* SRF deletion in vivo reduced *Gria2* mRNA level.

## Discussion

Describing the molecular basis of altered social behavior is essential in our understanding of developmental neuropsychiatric conditions like autism spectrum disorders or schizophrenia. Our study demonstrates that changes in gene regulation during early postnatal development obtained by the depletion of SRF, being one of the major transcriptional factors in the brain, exerts long-term consequences on the social behavior in adulthood. Moreover, we have shown that behavioral deficits in SRF KO mice are concomitant with alterations of dendritic spines morphology. Using primary neuronal cultures, we have got a mechanistic insight into the observed phenomena and unraveled SRF-dependent regulation of dendritic spine’s structure by affecting expression of glutamate receptors GluA1 and GluA2.

To address how disruption of general gene regulation during early postnatal development can affect social behavior in adulthood, we decided to knock-out SRF, a major transcriptional factor in the brain. SRF has been shown to control several immediate early genes, including *c-fos* and *Npas4* [22]. Additionally, single nucleotide polymorphisms of SRF’s coactivators – MKL1 and MKL2 have been found in patients with schizophrenia and autism spectrum disorders [34–36]. Using Cre-dependent SRF deletion at the early postnatal stage we avoided the detrimental effects of prenatal SRF elimination [10, 11, 16]. Such an experimental approach allowed us to study SRF role during critical period of neurodevelopment from a different perspective, namely to interfere with synapse maturation and evaluate the behavioral outcome during adulthood. Our study extends the results of Ramanan and colleagues [17] who deleted SRF later in postnatal development and characterized its function in the adult brain focusing on changes in gene expression and long-term potentiation.

To examine the behavioral phenotype, we used a novel, fully automated system, Eco-HAB^®^, that allowed us to assess animal behavior without human presence [21]. We measured social behavior of mice living in the groups simultaneously controlling for potentially interfering factors such as anxiety and locomotor deficits or diminished motivation [21]. The advantages of using Eco-HAB^®^ over standard sociability tests such as three-chamber apparatus include: ability to test multiple animals in one arena with individual identifying information, the ability to measure spontaneous and long lasting affiliations between mice, as well as a reduction in mice handling, which can significantly interfere with behavioral results. We found that both male and female SRF KO mice exhibited a highly specific deficit of interacting with well-known animals, in which they were less willing to spend time together with their co-housed conspecifics. SRF KO mice of both sexes were unable to establish and maintain social contacts, thus strengthening the relevance of the observed affiliative behavioral impairments. Similar deficiencies in sociability were identified in Fmr1 KO mice [21], a well-known model of autism [37, 38], as well as in most autistic patients [39]. Notably, our results showed no changes in social approach in male SRF KO mice, in contrast to female mice, in which we observed a slightly higher response to the novel olfactory cue. Sex-dependent behavioral disparities are often observed in developmental disorders, such as autism [40, 41], and the underlying causes require further investigation. Of note, previous studies exploring SRF role in the brain in adult animals (long after synaptic maturation is achieved), showed that SRF deletion, leads to alterations in rodent-typical behaviors such as nest building and marble burying [23].

The present study focused on the hippocampus, which is of particular interest when studying different aspects of interactions with familiar individuals [42]. Notably, hippocampal and social dysfunction oftentimes co-occur in multiple psychiatric disorders [43]. Hippocampal neural circuits have been considered critical for dynamic navigation through social contexts, including supporting social memory, tracking dynamic social behavior, and maladaptive interactions [44]. Moreover, hippocampus-dependent social memory is indispensable for the expression of the appropriate social behaviors and previous reports support that neural circuits in the ventral CA1 may be a locus of social memory engram [45]. All the aforementioned aspects are relevant to the reported impairments of sociability among familiar conspecifics, and indeed, may lay a foundation for the impaired in-cohort sociability caused by the SRF hippocampal knock-out.

However, we cannot exclude the involvement of other brain structures in the observed phenomena. Hippocampal neurons project to different brain areas that are critically engaged in the regulation of social behaviors [46], including the prefrontal cortex, amygdala, and anterior olfactory nucleus, the last having a strong influence on the olfactory bulb, which plays a role in social recognition [47]. Notably, the disruption of the OFC-dorsal hippocampus coupling was shown to impair social recognition [48]. However, our data show that deficits in voluntary interactions with the familiar conspecifics are not accompanied by the inability to recognize social stimuli, since SRF knock-outs readily respond to and explore novel social scents. Thus, the results support the dependence of the discovered deficits on the hippocampus alone, rather than on its impact on the function of the olfactory system.

Hippocampus-dependent behavioral deficits in sociability are often observed in several neurodevelopmental disorders, including ASD. The social deficits in SRF KO animals were accompanied by apparent dendritic spine dysmorphology manifested by their increased length and increased proportion of long, filopodia-like spines. Analogous changes, indicating general spine immaturity, were also shown in Fmr1 KO mice and patients with fragile X syndrome [49–51]. Of note, we did not observe any apparent malformations in hippocampal lamination, nerve fibers organization, or axon targeting deficits (Fig. S4), which could also account for the observed behavioral phenotype. Yet, we cannot completely exclude the existence of other structural changes (e.x. in the dendritic tree) that may contribute to social deficits in SRF KO mice.

In our experimental model, we diminished SRF expression in vivo at the early postnatal development, thus we preceded general spines structural and functional maturation which in rodents occurs in the first few weeks when abundant filopodia observed during the first postnatal week are gradually transformed into mature spines [52–55]. Thus, we were able to study long-term consequences of SRF-deficiency. To evaluate molecular components responsible for the observed behavioral and structural spine abnormalities, we used in vitro models of SRF depletion. It enabled us to further expand our research on dendritic spines, that harbor excitatory synapses and to get a comprehensive view of developmental SRF action in neurons. Moreover, hippocampal neurons in vitro are extensively used for morphological studies of spinogenesis because they pass through well-defined maturation stages over time [56]. In vitro experiments were designed, taking into consideration of potential non-specific effects of shRNAs, and applied scrambled shRNAs constructs as a control to activate RISC pathway also in control cells. In order to check whether morphological and biochemical changes in neurons are due to the lower SRF expression, we used validated shSRF construct [57]. Importantly, in our in vitro model we observed a significant rise in SRF transcriptional activity through the development. This result is consistent with already published in vivo data showing a sharp increase of SRF developmental expression level in the rat hippocampus and cortex [58], which further strengthens its importance in the early synapse development and neuronal maturation.

Additionally, we downregulated SRF in hippocampal cultures from *Srf*^*f/f*^ mouse, transduced or transfected with Cre-recombinase. Both in vitro strategies confirmed the immature morphology of dendritic spines observed in vivo, without changes in their density. Of note, a slight drop in dendritic spine number observed in mice cultures could be assigned to the applied experimental approach and Cre overexpression-related side effects.

We found that SRF, a primary regulator of activity-induced gene expression, was active during the specific time window of postnatal brain development and essential for the expression of mRNA for GluA1 (*Gria1*) and GluA2 (*Gria2*) in excitatory neurons in vitro. This was also confirmed in vivo, however, only for *Gria2*. The differences in the level of *Gria1* and *Gria2* mRNA expression in SRF depleted cells in vivo* vs. *in vitro could be explained by the fact that SRF is also expressed in astrocytes [59, 60], as well as both *Gria1* and *Gria2* mRNA [59–61]. Thus, the lack of Cre expression in astrocytes in vivo may lead to underestimation of the SRF-dependent changes in RNA levels. The observed dendritic spines juvenilization in SRF – depleted cells may be related to decreased GluA1 or GluA2 receptor level, since the number and composition of AMPARs at synapses determine their functional maturation and strength [62–65]. This statement is further supported by our functional studies showing the reduction of the amplitude of AMPAR-mediated mEPSCs recorded from SRF- lacking neurons. Our results are consistent with data that showed that the loss or overexpression of postsynaptic AMPARs altered overall synaptic transmission in developing neurons and affected synapse morphogenesis and maturation [65–67]. Interestingly, the lack of GluA2 expression decreased percentage of mushroom spines and increased amount of thin spines leading to spine maturation impairments [67, 68]. Since we did not observe gross changes in dendritic spine density in vivo, it seems that a certain level of GluA2 may be primarily required for spine stabilization and maintenance after synapse maturation is established [69]. Importantly it has been shown that AMPAR-dependent transmission can modulate social behaviors [70], and mutations and deletions of genes that encode AMPAR subunits, mainly GluA2, were recently linked to such neuropsychiatric conditions as ASD and mental disabilities [71, 72], thus may be responsible for the observed social deficits in SRF KO mice.

Our data suggest that SRF regulates filopodia transition into mature spines together with changes in the AMPARs level. Noteworthy, these two phenomena are well known to occur during dendritic spines' conversion to the one bearing functional synapses. Of note, the presented results and our previous discoveries on SRF function exclusively in adult neurons [23] provide a comprehensive picture of SRF as an essential regulator of dendritic spines morphology. Therefore, SRF expression is needed for both postnatal spine maturation and maintenance of the mature phenotype during the entire lifespan. The spine dysmorphology appear to directly translate into a reduction of the number of functional synapses, as we observed a decrease in the synaptic colocalization of well-known markers of excitatory synapses, Bassoon and PSD-95, in SRF-depleted neurons. This conclusion is further supported by the reduction of the mEPSCs frequency in shSRF neurons. Lower mEPSC frequencies, with no changes in dendritic spine density, suggest a role for SRF in their maturation into functional synaptic contacts.

Moreover, we showed the reduction of PSD-95 protein expression in SRF-depleted neurons. PSD-95 is a major postsynaptic scaffold protein that modulates postsynaptic function and the maturation of excitatory synapses [73, 74]. It is essential for AMPAR synaptic accumulation and AMPAR-mediated responses [75, 76]. Therefore the observed reduction of PSD-95- level in SRF-depleted neurons could also contribute to the smaller number of AMPARs at synapses. A significantly lower PSD-95 puncta number with no changes in Bassoon and increased percentage of immature spines might indicate that the overall number of synaptic connections is similar, but the number of functional/mature synapses is decreased.

Even though SRF was recently shown to bind *Gria1* and *Gria2* gene promoter regions in hippocampal neurons and mouse fibroblasts [7, 77, 78], we showed, for the first time, the downregulation of *Gria1* and *Gria2* mRNA level in neurons lacking SRF, however we do not exclude the possible, indirect impact of other transcription factors, SRF targets, on the regulation of AMPAR expression [79, 80]. In particular, we have previously demonstrated that SRF regulates the expression of c-*fos*, a component of the AP-1 transcription factor complex [22] which is a crucial mediator of postnatal neuronal development [81]. Our results are especially significant in the context of a recent study by Del Blanco et al. (2019), who investigated the role of cyclic adenosine monophosphate response element-binding protein (CREB)-binding protein (CBP) in spine maturation [7]. The lack of CBP during neuronal development alters the expression of genes involved in neuronal growth and plasticity that have SRF binding elements. The deficits in dendritic spine maturation and activity-dependent synaptic remodeling in CBP-lacking neurons were reversed by the overexpression of SRF transcription through an SRF-VP16 construct [7]. Several lines of evidence suggest that SRF and CBP may co-regulate gene expression [82–84]. The morphological, biochemical, and physiological synaptic immaturity shown in our study may be a direct consequence of a decrease in the chromatin occupancy of SRF, the lack of its interaction with CBP, or an indirect consequence of SRF-regulated c-*fos* expression in postnatal neurons.

In our study we revealed that early postnatal SRF expression in excitatory neurons is a potent regulator of different aspects of cohort mice sociability and we report on the underlying neurobiological mechanism that contributes to behavioral abnormalities in SRF KO mice. Molecular and behavioral phenotype described in SRF-deficient mice shares similar characteristics with ASD mice models. Altogether, structural and functional alterations of dendritic spines observed in SRF-depleted neurons implicate excitatory synapses as crucial substrates of pathogenesis in different neurodevelopmental disorders. The identification of novel molecular pathways that are involved in the regulation of aspects of sociability may reveal the neuronal mechanisms that underlie some neuropsychiatric diseases, such as ASD.

### Supplementary Information

Below is the link to the electronic supplementary material.

Supplementary file1 (PDF 34745 KB)

## Data Availability

All data are available in the main text or the supplementary materials.
