# Supplementary material for: SRF depletion in early life contributes to social interaction deficits in the adulthood
Source: Cell Mol Life Sci. 2022 May 4;79(5):278. doi: 10.1007/s00018-022-04291-5 (PMC9064851; doi:10.1007/s00018-022-04291-5)
Supplement: Supplementary file 1 — Supplementary file1 (PDF 34745 KB) [file 18_2022_4291_MOESM1_ESM.pdf]

Cellular and Molecular Life Sciences

Title: SRF depletion in early life contributes to social interaction deficits in the adulthood

Authors: Matylda Roszkowska[1]†, Anna Krysiak[1]†, , Lena Majchrowicz[1], Karolina Nader[1], Anna Beroun[2], Piotr Michaluk[1], Martyna Pekala[1], Jacek Jaworski[3], Ludwika Kondrakiewicz[4], Alicja Puścian[4], Ewelina Knapska[4], Leszek Kaczmarek[1] and Katarzyna Kalita[1]*

[1]Laboratory of Neurobiology, Nencki-EMBL Partnership for Neural Plasticity and Brain Disorders – BRAINCITY, Nencki Institute of Experimental Biology, Polish Academy of Sciences, 3 Pasteur Street, 02-093 Warsaw, Poland

*Corresponding author: **k.kalita@nencki.edu.pl**, Researcher ID: R-4072-2016

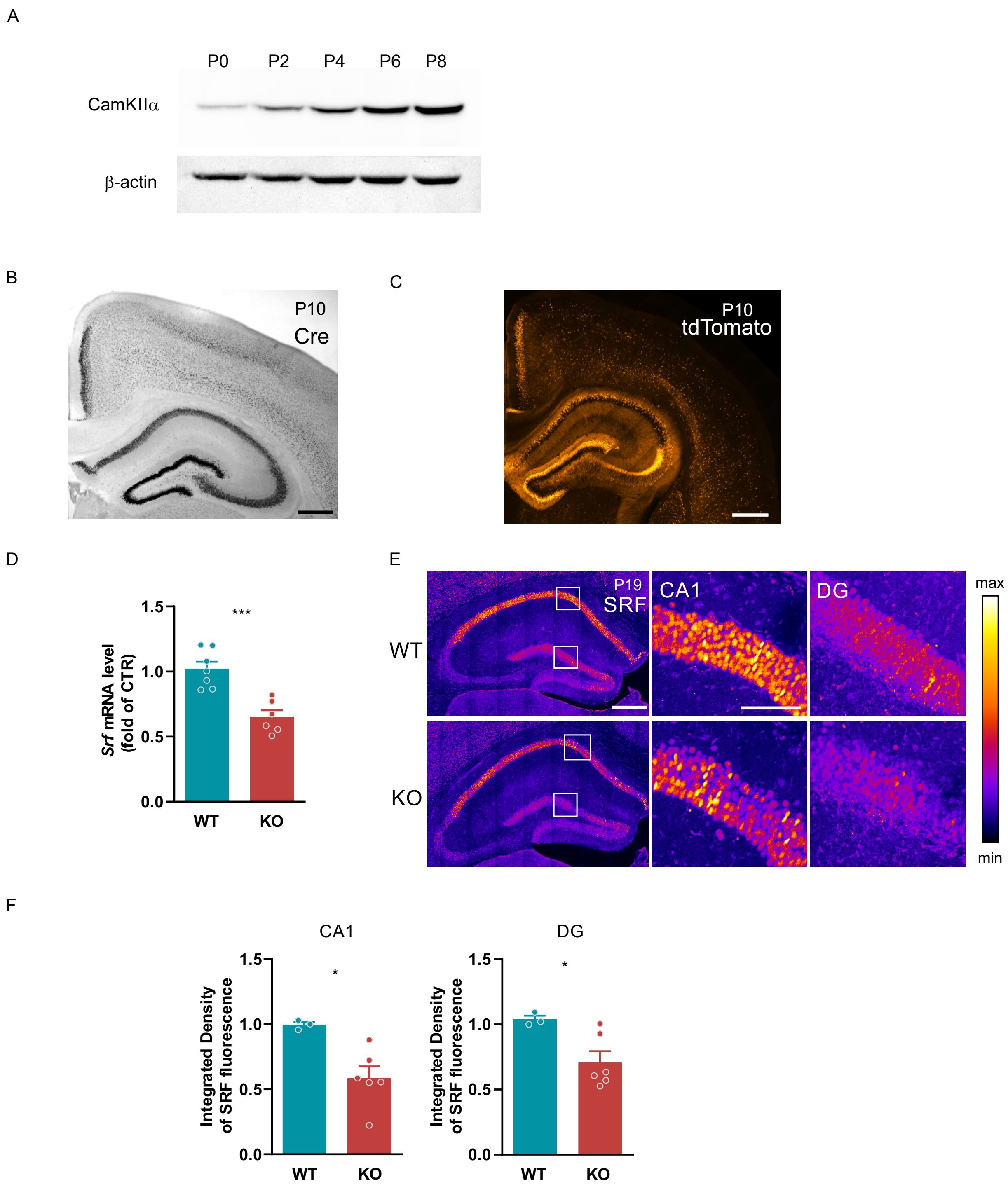

Fig. S1. Model validation of SRF deletion *in vivo* induced by 4-Hydroxytamoxifen injections. (a) Western blot of CaMKIIα expression in the mouse hippocampus during postnatal development (P0-P8). **(b)** Example image of nuclear CaMKCreERT2-Cre recombinase expression in the brain of the transgenic mice at P10 after three injections of 4-OHT (at P5, 7 ,9) visualized by immunohistochemical staining using anti-Cre Ab. **(c)** Example image of tdTomato labeled neurons at the brain slice from *loxP*-flanked STOP tdTomato^{CaMKCreERT2} transgenic mice at P10 after three injections of 4-OHT (at P5, 7 ,9). **(d)** Analysis of Srf knock-out efficiency by 4-OHT injections in young, P12-14 mice hippocampus. WTn_{mice} = 7; KOn_{mice} = 6. **(e)** Conditional deletion of SRF during development analyzed in the young mice hippocampus at P20. Immunofluorescent staining of SRF in false image color in CTR and SRF KO animals. SRF elimination can be observed in the dentate gyrus (DG), and CA1 subfield of the hippocampus. White squares correspond to enlarged images of CA1 and DG. The corresponding color histogram depicts the SRF fluorescence intensity (*min - max*) in a false-color scheme ('*Fire* LUT'). Data in (d) are presented as a fold change relative to control. Data in (f) were normalized to control. Student's *t*-test in all data. Data as means ± SEM. *$p < 0.05$, ***$p < 0.001$. Scale bars (b,c,e) = 400 µm; (b, enlarged images = 100 µm).

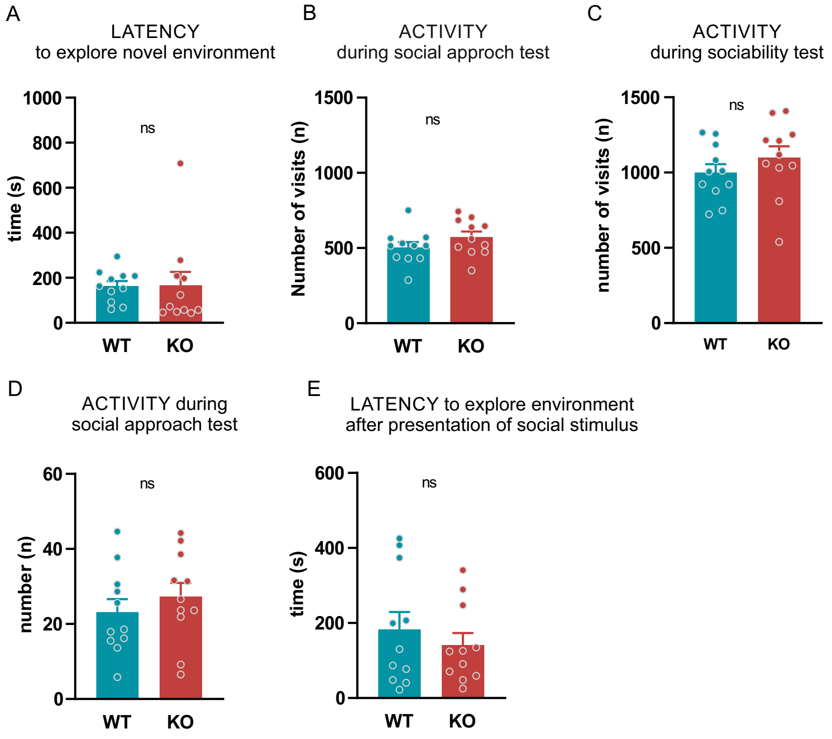

Fig. S2. Various behavioral aspects measured during sociability and social approach tests in male mice. (a) WT and SRF KO mice do not differ in the anxiety level, measured as latency to explore the entire novel environment. WT and SRF KO mice show similar locomotor activity levels during both **(b)** social approach test and **(c)** sociability test. **(d)** WT and SRF KO mice do not differ in their ability to follow each other and thus gather information from conspecifics during social approach tests. Followings are measured as a number of events when one mouse followed another through any of the corridors of the Eco-HAB® system. **(e)** WT and SRF KO are equally efficient in scouting environment during social approach test and thus gaining information about the appearance of novel olfactory stimuli, as measured by the latency to explore each compartment of the Eco-HAB® after the presentation of social and non-social scents. WT $n_{mice} = 11$; KO $n_{mice} = 11$. Student's t-test in all data. Data as means ± SEM; $^{ns}p > 0.05$.

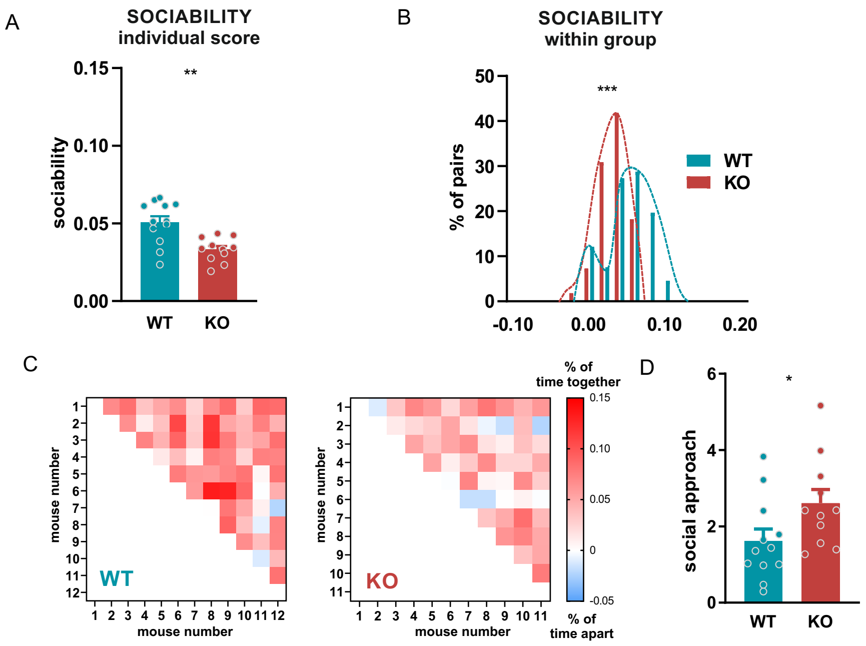

Fig. S3. SRF KO female mice exhibit decrease in sociability, similar to males. (a) Sociability, measured as the time voluntarily spent together with others, in WT and SRF KO mice. **(b)** Histogram of the distribution of in-cohort sociability in WT and SRF KO mice**. (c)** Data from **(a)** and **(b),** represented as a matrix in which squares illustrate the time voluntarily spent together by each pair of mice within the cohort. The intensity of colors reflects the strength of the relationship in accordance with the presented scale. **(d)** Social approach in WT and SRF KO mice. WTn_{mice} = 12; KOn_{mice} = 11. Student's t-test in **(a,d)** and Kolmogorov-Smirnov test **(b).** Data as means ± SEM. *$p < 0.05$, **$p < 0.01$, ***$p < 0.001$.

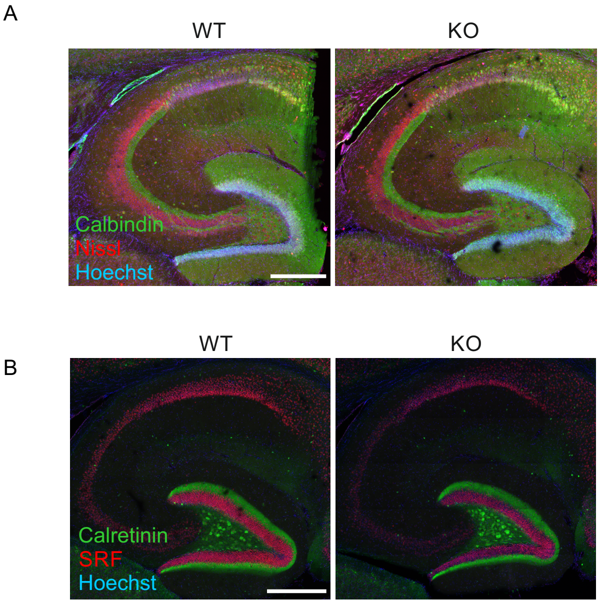

Fig. S4. Early postnatal SRF deletion does not disrupts hippocampal lamination and axon pathfinding. **(a)** Representative images from WT and SRF KO adult mice slices stained with calbindin antibody (green), Nissl NeuroTrace (red) and Hoechst 3334 (blue). **(b)** Representative images from WT and SRF KO adult mice slices stained with calretinin antibody (green), SRF (red) and Hoechst 3334 (blue). Scale bars (a, b) = 400 µm.

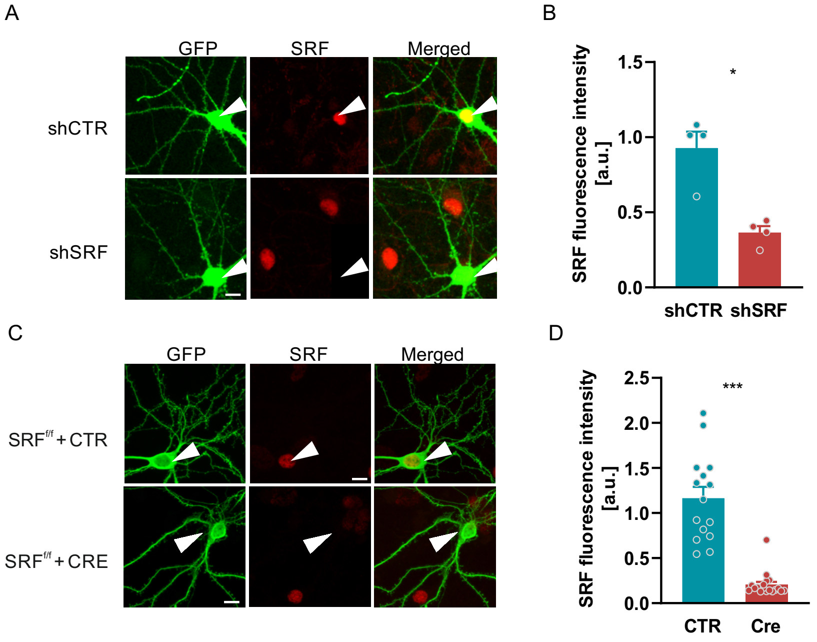

Fig. S5. SRF knockdown in primary hippocampal neurons reduced SRF expression *in vitro*. (a, c) Representative images of **(a)** GFP-positive rat hippocampal neurons that were transfected with shCTR (control) or shSRF plasmid and **(c)** $SRF^{f/f}$ mouse hippocampal neurons that were co-transfected with CaMKIIα-GFP (control) together with CaMKIIα-Cre-recombinase or CaMKIIα-GFP alone and immunostained against endogenous SRF (red). The white arrowheads indicate transfected cells. **(b)** The shSRF and **(d)** CamKIIα-Cre mediated SRF knockdown efficiency estimated by the average intensity of the SRF immunofluorescence (IF) signal in transfected cells (at single cell level). shCTRn_{cells}= 4; shSRFn_{cells}= 4 in **(b)** and CTRn_{cells} = 15; Cre n_{cells} = 18 **(d)**. Mann-Whitney test in **(b,d)**. Data as means ± SEM. *$p < 0.05$, ***$p < 0.001$. Scale bars (a, c) = 10 µm.

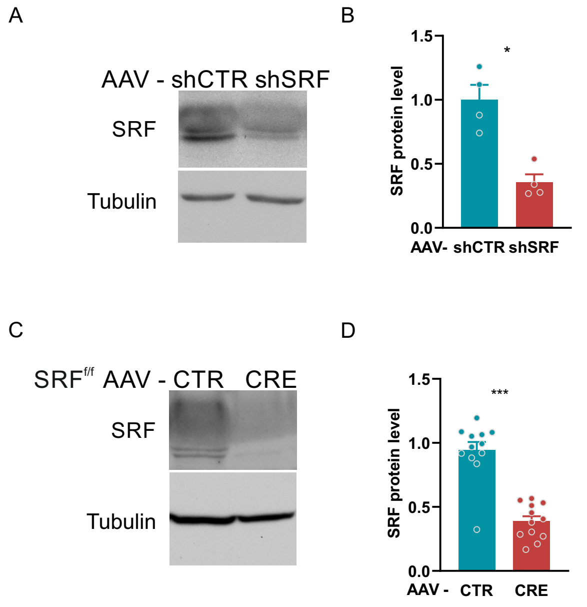

Fig. S6. AAV-mediated transduction of primary hippocampal neurons downregulates SRF expression. (a, b) Western blots of protein extracts from **(a)** AAV-shCTR or AAV-shSRF-transduced rat hippocampal neurons and **(c)** AAV-CAMKIIα-mCherry or AAV-CAMKIIα-Cre- transduced Srf$^{f/f}$ mouse hippocampal neurons. **(b, d)** Quantification of SRF total protein levels in AAV-shCTR- and AAV-shSRF transduced- rat hippocampal neurons (b) and AAV-CAMKIIα-mCherry- and AAV-CAMKIIα-Cre - transduced Srf$^{f/f}$ mouse hippocampal neurons **(d).** All of the data are presented as a fold change relative to control. AAV-shCTRn_{reps}= 4; AAV-shSRFn_{reps}= 4 in (b) and AAV-CTRn_{reps} = 12; AAV-Cren_{cells} = 12 **(d).** Mann-Whitney test **(b,d).** The data from rat hippocampal cultures in **(b)** passed the normality test. $^{**}p = 0.0028$ (Student's t-test). Data as means ± SEM. $^{*}p < 0.05$, $^{***}p < 0.001$.

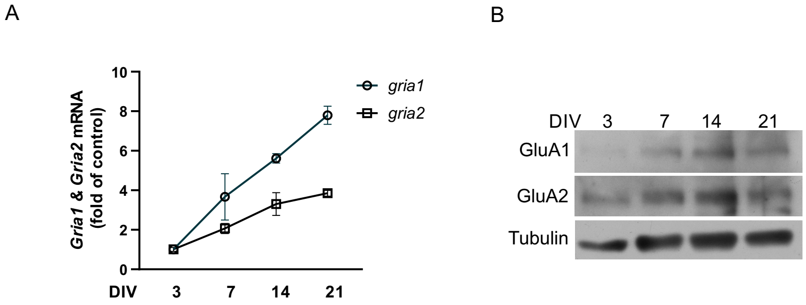

Fig. S7. Developmental changes of GluA1 and GluA2 protein expression *in vitro.* (a) Changes in *Gria1* and *Gria2* mRNA level in rat primary hippocampal cultures on DIV3, 7, 14, and 21. **(b)** Western blots of protein extracts collected from rat hippocampal cultures at DIV3, 7, 14, and 21. Data as means ± SEM.
